# Association of cardiovascular health and periodontitis: a population-based study

**DOI:** 10.1186/s12889-024-18001-2

**Published:** 2024-02-12

**Authors:** Haitao Qu, Shengnan Zhang

**Affiliations:** Department of Oral and Maxillofacial Surgery, Jinan Stomatological Hospital, Jinan, 250001 China

**Keywords:** Periodontitis, Cardiovascular health, CVD, Life's essential 8, Socioeconomic status

## Abstract

**Background:**

There is a strong association between cardiovascular disease (CVD) and periodontitis. This study utilized the Life Essentials 8 (LE8) score, a composite measure of cardiovascular health (CVH), to elucidate the relationship between CVH and periodontitis.

**Methods:**

Data from 8,649 nationally representative participants in the National Health and Nutrition Examination Survey (NHANES) were analyzed. The independent variable in our study was the CVH score (a higher CVH score indicates better cardiovascular health), and the dependent variable was the presence or absence of periodontitis. The association between CVH and periodontitis was investigated using weighted multivariable logistic regression models and restricted cubic spline (RCS). We controlled for potential confounders such as age, sex, race, education, and socioeconomic status to minimize bias.

**Results:**

There was a negative association between the total CVH score and the odds of periodontitis. After adjusting for all covariates, a 10-point increase in total CVH score was associated with a 10% lower in the odds of periodontitis [0.90 (0.87, 0.93)]. Participants with a higher CVH had 40% lower odds of periodontitis compared with those with a lower CVH. Socioeconomic status (education and income) modified this association (*P* for interaction < 0.05).

**Conclusion:**

Our study suggests that better cardiovascular health, as indicated by higher CVH scores, is associated with a reduced likelihood of periodontitis among US adults. The relationship between CVH and periodontitis appears to be influenced by socioeconomic status, emphasizing the need for targeted interventions in populations with lower socioeconomic status.

**Supplementary Information:**

The online version contains supplementary material available at 10.1186/s12889-024-18001-2.

## Introduction

Cardiovascular health (CVH), a critical aspect of global health, is increasingly recognized for its interconnectedness with various aspects of systemic health, including oral health [1]. The American Heart Association (AHA) introduced the Cardiovascular Health (CVH) concept in 2010, initially measured by the Life’s Simple 7 (LS7) score. This score encompassed seven crucial health behaviors and factors [1]. In 2022, the AHA updated this to the Life’s Essential 8 (LE8), incorporating sleep health and refining its assessment algorithm in response to evolving research and a broadened understanding of CVH [2–4]. The LE8 score, now a more encompassing and precise tool, allows for a detailed evaluation of CVH and its influence on health outcomes.

Emerging evidence indicates a bidirectional relationship between periodontal disease and cardiovascular health [5–7]. Periodontitis, a prevalent inflammatory condition affecting the supporting structures of teeth, has been shown to have potential implications for cardiovascular health [8]. Recent studies have increasingly highlighted the significant influence of cardiovascular health on periodontal disease. Periodontitis, a chronic inflammatory condition affecting the supporting structures of teeth, is not only a consequence of local factors but is also impacted by systemic health conditions, particularly cardiovascular diseases (CVD). There is a growing body of evidence suggesting that cardiovascular diseases exacerbate periodontal conditions [9]. For instance, systemic inflammation, a common factor in CVD, has been shown to play a major role in the progression of periodontal disease, suggesting a close interplay between the two factors [10]. This connection is further supported by studies demonstrating that conditions such as atherosclerosis, often associated with poor CVH, are significantly linked with the severity of periodontitis [11]. Furthermore, the inflammatory burden imposed by periodontal disease may also contribute to the worsening of cardiovascular conditions, underscoring the bidirectional nature of this relationship [12].

Recognizing this intricate relationship, our study aims to elucidate the association between comprehensive cardiovascular health, as quantified by the LE8 metrics, and periodontal outcomes. By leveraging NHANES data, this study seeks to contribute to the understanding of how cardiovascular wellbeing, encompassing a broader range of health metrics, correlates with periodontitis.

## Methods

### Study population and design

**Fig. 1:**
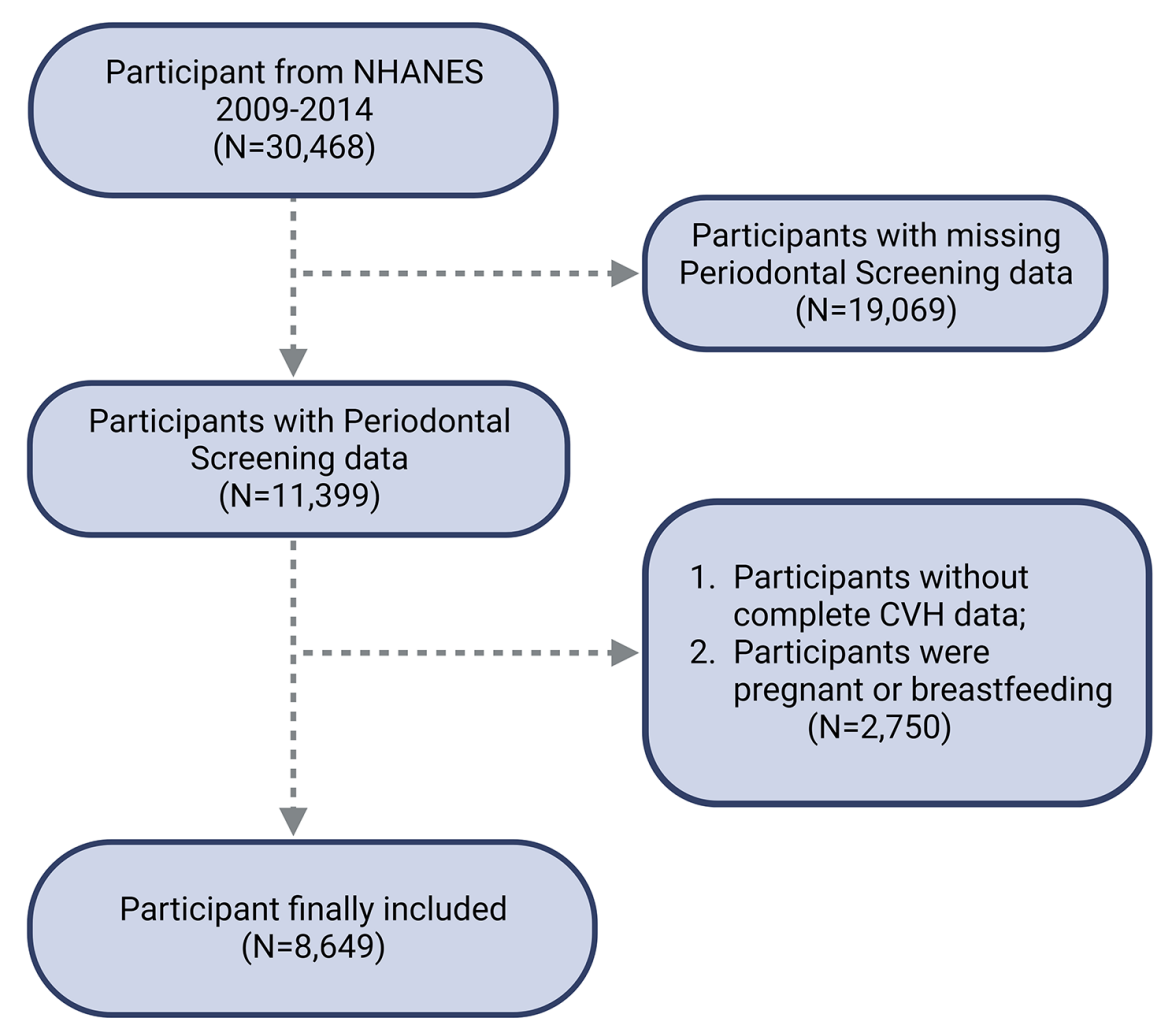
Flow chart of participants selection. NHANES, National Health and Nutrition Examination Survey

The National Health and Nutrition Examination Survey (NHANES), facilitated by the National Center for Health Statistics (NCHS), functions as a comprehensive, cross-sectional study aimed at assessing the health and nutritional status of the U.S. civilian population [13, 14]. Initiated in 1999, NHANES employs a diverse methodology that includes physical examinations and interviews to collect data on demographics, socioeconomic factors, and health indicators [15]. Comprehensive descriptions of the study’s design and methods are available on the NHANES website. The present research analyzes data from multiple NHANES cycles spanning 2009–2014. Strict exclusion criteria were applied, omitting 19,069 individuals lacking complete periodontal screening data and 2,750 individuals with insufficient information to compute Cardiovascular Health (CVH) metrics or who were pregnant during the baseline examination (Fig. 1). This resulted in a final analytical sample of 8,649 participants. The participant selection process adhered to ethical standards, and the survey protocol received approval from the NCHS Research Ethics Review Board. All participants provided written informed consent, and this research complies with the Strengthening the Reporting of Observational Studies in Epidemiology (STROBE) reporting guideline.

### Cardiovascular health evaluation

Cardiovascular Health (CVH) is assessed using the LE8 score, a higher CVH score indicates better cardiovascular health, which amalgamates eight crucial elements: diet, physical activity, use of tobacco or nicotine, sleep habits, Body Mass Index (BMI), non-HDL cholesterol, blood glucose levels, and blood pressure metrics. The diet component is evaluated using the Dietary Approaches to Stop Hypertension (DASH) diet score. Information regarding physical activity (quantified as minutes of moderate to vigorous weekly exercise), tobacco or nicotine usage (including active smoking and exposure to secondhand smoke), sleep habits (quantified as total hours of sleep), and medication usage were obtained from standardized questionnaires. Parameters such as weight, height, blood glucose levels, and blood pressure were gathered at specialized mobile centers following standardized protocols. BMI is calculated by dividing an individual’s weight in kilograms by the square of their height in meters. The average blood pressure was determined from all readings taken during the initial evaluation. Serum cholesterol levels were ascertained using enzymatic techniques, and non-HDL cholesterol was determined by subtracting HDL cholesterol from the total cholesterol value. Glycated hemoglobin levels were assessed using high-performance liquid chromatography. Detailed descriptions and scoring methodologies for each CVH component are available in Table S1 and the cited studies [2, 3, 16]. Each CVH metric is scored on a scale from 0 to 100. The overall CVH score is computed as the average for the eight individual factors. According to the American Heart Association guidelines, CVH scores of 80–100, 50–79, and 0–49 correspond to high, moderate, and low CVH levels, respectively [17].

### Definition of periodontitis

In the NHANES data, periodontal examinations were conducted on both males and females aged 30 years and above. The “Oral Health - Periodontal Screening” initiative evaluates periodontal health by measuring six regions of up to 28 teeth. The program utilizes two clinical metrics: Clinical Attachment Loss (CAL) and Probing Depth (PD). A classification system for periodontitis, based on case definitions from the CDC and the American Academy of Periodontology (AAP), has been established. Severe periodontitis is identified by at least two interproximal areas with a CAL of ≥ 6 mm and at least one interproximal area with a PD of ≥ 5 mm, not on the same tooth. Moderate periodontitis is defined by two or more interproximal sites with a probing pocket depth of ≥ 5 mm or a clinical attachment level of ≥ 4 mm, not on the same tooth [18]. Cases of moderate to severe periodontitis are classified as patients with periodontitis, while all other cases are categorized as the reference group [19].

### Covariables

Data about demographics, including age, gender, ethnic background, and educational attainment, was gathered using standardized surveys. The classifications for ethnicity encompassed White, Black, Mexican American, other Hispanic, and another category that included other races such as Asian and multiracial individuals. Educational attainment was segmented into three categories: those without a high school diploma, graduates, and individuals with some college education or higher. The ratio of family income to the poverty threshold was determined by dividing the total family income by the official poverty line and subsequently categorized into low (< 1.3), intermediate (≥ 1.3 and < 3.0), and high (≥ 3.0) groups [15]. The selection of these covariates complements the CVH score components, which already include information on diet quality, physical activity duration, smoking status, sleep duration, body mass index, blood lipids, blood glucose, and blood pressure. This approach ensures a comprehensive adjustment for potential confounders in our analysis, aligning with methodologies applied in similar high-quality studies [2, 20, 21]. Our covariate selection process and model adjustments are grounded in the CVH’s encompassing nature, as these demographic and socioeconomic factors provide additional insights beyond the CVH components.

### Statistical analysis

To enhance representativeness at the national level, NHANES sampling weights were applied, compensating for demographic overrepresentation and complex survey design [22–24]. CVH was categorized as low (< 50), intermediate (50–79), and high (≥ 80) based on the LE8 score. Categorical variables were expressed as percentages (weighted counts), and continuous variables were denoted as mean ± standard deviation (SD). Multiple imputation techniques were employed to address missing covariate data. Logistic regression models were used to investigate the relationships between the overall CVH score, its 8 subscores, and periodontitis. Three regression models were employed: Model 1, which was unadjusted for covariates (but adjusted for the other 7 subscores when assessing CVH subscores); Model 2, which built upon Model 1 by incorporating age, sex, and ethnicity; and Model 3, which extended Model 2 by adding education level and family income-to-poverty ratio. The association between CVH and periodontitis was also analyzed across various subgroups, includes sex (male/female), race (Non-Hispanic White/ Non-Hispanic Black/ Mexican American/ Other Hispanic/ Others), education level (Less than high school/ High school/ More than high school), and PIR categories (< 1.3/1.3–3.5/>3.5). To explore potential non-linear relationships between CVH and periodontitis, a restricted cubic spline (RCS) was utilized. All statistical analyses were performed using R software version 4.3.0. All tests were two-sided, with a *P* value < 0.05 indicating statistical significance.

## Results

### Baseline characteristics

**Fig. 2:**
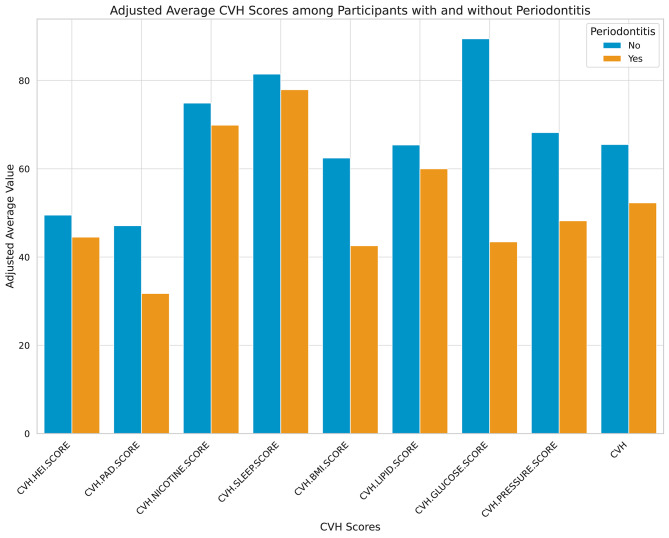
Distribution of Life’s Essential 8 total score and 8 sub-scores in participants with and without periodontitis. Note: a higher CVH score indicates better cardiovascular health

In the present analysis, a total of 8,649, approximating 50.1 million U.S. adults were included. The mean (SD) for the LE8 score was 65.75 (12.46), and the odds of periodontitis was 49.8 (24.9). Figure 2 depicts the distribution of the CVH total score and 8 subscores among participants with or without periodontitis. The participant distribution, as determined by their CVH status (LE8 score), was as follows: 15.9% (equating to 8.0 million individuals) exhibited low CVH (LE8 < 50), 62.6% (approximating 31.3 million individuals) had moderate CVH (50 ≤ LE8 < 80), and 21.5% (corresponding to 10.8 million individuals) were classified with high CVH (LE8 ≥ 80). Initial evaluations revealed that those participants categorized with high CVH were generally younger and, more frequently, women and White. They were also more likely to possess a higher level of educational achievement and PIR. Additionally, these individuals exhibited a lower odd of periodontitis, as detailed in Table 1.

**Table 1:** Characteristics of US adults by three categories of total cardiovascular health (CVH) score, 2005–2018

Characteristics	Total	Low (LE8 < 50)	Moderate(50 ≤ LE8 < 80)	High (LE8 ≥ 80)
Prevalence, % (weighted *N*, in millions)	100 (50.1)	15.9 (8.0)	62.6 (31.3)	21.5 (10.8)
No. of participants in sample	8,649	1,375	5,414	1,860
Age, years	51.80 ± 12.35	53.12 ± 12.58	51.84 ± 11.26	46.53 ± 12.45
Female, % (weighted *N*, in millions)	50.3 (25.2)	45.6 (3.6)	48.1 (15.1)	60.3 (6.5)
Race/ethnicity, % (weighted *N*, in millions)				
Non-Hispanic White	64.7 (32.4)	63.2 (5.1)	63.5 (19.9)	69.7 (7.5)
Non-Hispanic Black	10.6 (5.3)	15.1 (1.2)	11.2 (3.5)	5.0 (0.1)
Mexican American	9.2 (4.6)	10.5 (0.8)	9.5 (3.0)	7.5 (0.8)
Other Hispanic	6.4 (3.2)	5.1 (0.4)	6.5 (2.0)	6.7 (0.7)
Others	9.1 (4.6)	6.1 (0.5)	9.3 (2.9)	11.1 (1.1)
Education level, % (weighted *N*, in millions)				
Less than high school	12.3 (6.2)	19.7 (1.6)	12.6 (3.9)	5.8 (0.6)
High school	23.5 (11.8)	32.7 (2.6)	25.3 (7.9)	11.1 (1.2)
More than high school	64.2 (32.1)	47.6 (3.8)	62.1 (19.5)	83.1 (9.0)
PIR, % (weighted *N*, in millions)				
Low (< 1.3)	20.8 (10.4)	30.9 (2.5)	20.0 (6.3)	13.9 (1.5)
Intermediate (1.3–3.5)	27.0 (13.5)	30.2 (2.4)	28.2 (8.8)	21.6 (2.3)
High (> 3.5)	52.2 (26.2)	38.9 (3.1)	51.8 (16.2)	64.5 (7.0)
Periodontitis, % (weighted *N*, in millions)	49.8 (24.9)	65.9 (5.3)	49.6 (15.5)	38.0 (4.1)
AHA LE8 score (SD)				
Mean total CVH score	65.75 (12.46)	42.26 (5.42)	63.63 (6.97)	86.35 (5.47)
Mean DASH diet score	42.31 (30.07)	23.04 (21.05)	40.51 (30.48)	65.15 (28.09)
Mean physical activity score	51.47 (43.02)	9.14 (24.79)	49.67 (46.45)	88.52 (27.65)
Mean tobacco exposure score	68.50 (31.31)	45.23 (33.93)	67.62 (32.29)	90.81 (19.70)
Mean sleep health score	85.16 (20.62)	73.51 (22.39)	84.45 (19.91)	92.64 (14.95)
Mean body mass index score	57.18 (29.36)	31.05 (24.40)	54.64 (30.23)	86.39 (20.13)
Mean blood lipid score	67.81 (24.49)	48.13 (30.73)	66.44 (25.30)	87.51 (21.11)
Mean blood glucose score	83.12 (21.46)	66.38 (30.45)	85.55 (22.93)	98.14 (8.54)
Mean blood pressure score	64.77 (27.83)	42.61 (25.53)	67.16 (25.43)	88.83 (17.83)

Mean (SD) for continuous variables: the *P* value was calculated by the weighted linear regression model

Percentages (weighted N, in millions) for categorical variables: the *P* value was calculated by the weighted chi-square test

Cardiovascular health (CVH) is categorized into 3 grades,low:LE8 score < 50, medium:50 ≤ LE8 score < 80, high:LE8 score ≥ 80

Abbreviation: AHA, American Heart Association; LE8, Life’s Essential 8; CVH, cardiovascular health; PIR. Ratio of family income to poverty; DASH, Dietary Approaches to Stop Hypertension

Note: a higher CVH score indicates better cardiovascular health

### Association of the CVH and periodontitis

**Fig. 3:**
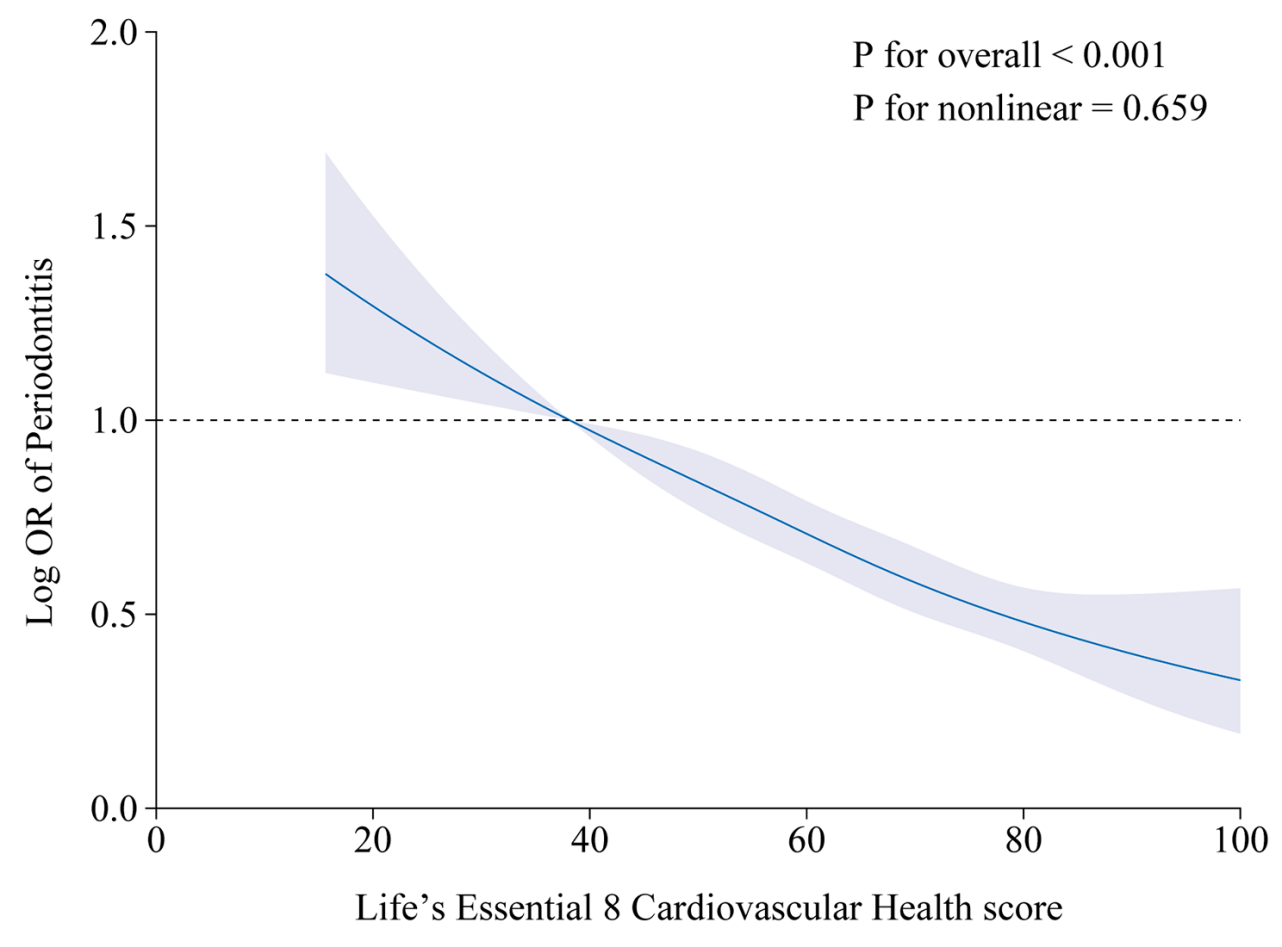
Dose–response relationships between the life’s essential 8 total score and periodontitis. Beta (solid lines) and 95% confidence levels (shaded areas) were adjusted for age, gender, race, education level, and ratio of family income to povert. Note: a higher CVH score indicates better cardiovascular health

Table 2 demonstrates the association of the total CVH score and the eight CVH subscores with periodontitis. In all models, there was a negative correlation between the total CVH score and the odds of periodontitis. After adjusting for all covariates, a 10-point higher CVH score was associated with a 9% lower odds of periodontitis prevalence [0.90 (0.87, 0.93)]. The odds of periodontitis lower by 25% in participants with moderate CVH scores [0.75 (0.60, 0.95)] and by 40% in those with high CVH scores [0.60 (0.41, 0.89)], compared with participants with low CVH scores. In addition, in the fully adjusted model, all six CVH subscores maintained negative associations with periodontitis, except for the Physical activity score and Blood lipid score, which had non-significant positive associations with the odds of periodontitis. Figure 3 further demonstrates the negative correlation association between the total CVH score and the odds of periodontitis (*P* for overall < 0.001; *P* for non-linear = 0.659).

**Table 2:** Adjusted odds ratios of life’s essential 8 cardiovascular health (CVH) score and periodontitis, NHANES 2005–2018

CVH components (per 10 scores)	Model 1^a^ [OR (95% CI)]	Model 2^b^ [OR (95% CI)]	Model 3^c^ [OR (95% CI)]
**Total CVH score**	0.79 (0.77, 0.82)	0.84 (0.82, 0.87)	0.90 (0.87, 0.93)
Low (LE8 < 50)	1 (ref.)	1 (ref.)	1 (ref.)
Moderate (50 ≤ LE8 < 80)	0.73 (0.58, 0.92)	0.78 (0.62, 0.98)	0.75 (0.60, 0.95)
High (LE8 ≥ 80)	0.57 (0.40, 0.80)	0.62 (0.44, 0.88)	0.60 (0.41, 0.89)
**Subgroup CVH scores**			
DASH diet score	0.94 (0.92, 0.96)	0.94 (0.91, 0.96)	0.96 (0.92, 0.99)
Physical activity score	1.03 (0.98, 1.08)	1.02 (0.99, 1.05)	1.01 (0.99, 1.03)
Tobacco exposure score	0.95 (0.91, 0.99)	0.97 (0.95, 0.99)	0.97 (0.94, 0.99)
Sleep health score	0.99 (0.95, 1.02)	0.98 (0.94, 1.01)	0.98 (0.94, 1.01)
Body mass index score	0.95 (0.92, 0.98)	0.95 (0.92, 0.98)	0.95 (0.92, 0.98)
Blood lipid score	0.98 (0.95, 1.01)	1.00 (0.97, 1.04)	1.00 (0.98, 1.02)
Blood glucose score	0.92 (0.89, 0.95)	0.95 (0.91, 0.98)	0.94 (0.91, 0.97)
Blood pressure score	0.94 (0.90, 0.98)	0.96 (0.91, 1.01)	0.98 (0.95, 1.01)

^a^Model 1: no covariates were adjusted

^b^Model 2: age, gender, and race were adjusted

^c^Model 3: age, gender, race, education level, and ratio of family income to poverty were adjusted

Abbreviation: CI, confidence interval; CVH, cardiovascular health; DASH, Dietary Approaches to Stop Hypertension

Note: a higher CVH score indicates better cardiovascular health

### Subgroup analyses

To investigate whether the association between CVH and periodontitis differed across populations, subgroup analyses based on sex, race, and socioeconomic status (education and PIR) were further investigated (Table 3). In all subgroups, the negative association between total CVH and periodontitis prevalence was maintained (*P* < 0.05). Nevertheless, the results showed significant differences in the association between CVH and periodontitis among different socioeconomic status groups (*P* for interaction < 0.05). A 10-point increase in total CVH score was associated with a 22% lower in the odds of periodontitis among participants with less than high school education [0.78 (0.69, 0.87)], which was significantly higher than among those with high school education [0.90 (0.86, 0.95)] and those with more than high school education [0.88 (0.80, 0.96)]. The same was true for household income, where a 10-point increase in total CVH score was associated with 28% lower odds in periodontitis among participants with lower PIR [0.72 (0.64, 0.80)], significantly higher than among participants with moderate [0.85 (0.79, 0.91)] and higher PIR [0.92 (0.86, 0.98)].

**Table 3:** Subgroup analysis of the association between between the life’s essential 8 cardiovascular health score and periodontitis

Subgroup	OR (95%CI)	*P* for interaction
**Sex**		0.425
Male	0.87 (0.78, 0.96)	
Female	0.91 (0.83, 0.99)	
**Race/ethnicity**		0.228
Mexican American	0.82 (0.63, 1.01)	
Other Hispanic	0.78 (0.63, 0.93)	
Non-Hispanic White	0.92 (0.86, 0.98)	
Non-Hispanic Black	0.86 (0.71, 1.03)	
Other races	0.86 (0.64, 1.00)	
**Education level**		0.036
Less than high school	0.78 (0.69, 0.87)	
High school	0.90 (0.86, 0.95)	
More than high school	0.88 (0.80, 0.96)	
**PIR**		0.025
Low (< 1.3)	0.72 (0.64, 0.80)	
Intermediate (1.3–3.5)	0.85 (0.79, 0.91)	
High (> 3.5)	0.92 (0.86, 0.98)	

Age, gender, race, education level, and ratio of family income to poverty were adjusted

Abbreviation: CI, confidence interval; CVH, cardiovascular health; DASH, Dietary Approaches to Stop Hypertension

Note: a higher CVH score indicates better cardiovascular health

## Discussion

In this nationally representative study, we found a significant negative correlation between cardiovascular health, as quantified by the LE8 score, and the odds of periodontitis. More importantly, our results suggest that differences in socioeconomic status modify the association between CVH and periodontitis, with individuals of lower socioeconomic status benefiting more from higher CVH scores. These findings emphasize the potential impact of cardiovascular health on the odds of periodontitis and highlight the importance of CVH scores quantified by LE8 in monitoring and reducing the odds of periodontitis.

To the best of our knowledge, this is the first study to investigate the relationship between exposure to CVH scores and periodontitis. Previous relevant studies have focused on the association between specific cardiovascular diseases and periodontitis [25]. In a recent meta-analysis study, in which 30 prospective cohort studies were included, the results showed a significantly higher cardiovascular risk of 20% in patients with periodontitis compared to participants without periodontitis (RR = 1.20,95% CI 1.14–1.26) [26]. A large case-control study from Sweden showed a significant 28% increase in the incidence of myocardial infarction in participants with periodontitis after detailed adjustment for all confounding factors [27]. However, it is not enough to understand the association between CVD and periodontitis; the pathogenesis of periodontitis is also strongly associated with a variety of cardiovascular risk factors, and the CVH score measured by LE8 in this study encompassed the common risk factors shared by periodontitis and cardiovascular risk. For example, smoking increases the risk of periodontitis and atherosclerosis and has a strong negative impact on the response to periodontal therapy [28]. In addition, periodontitis is associated with variables in plasma lipid levels, and abnormalities in blood lipids will accelerate disease progression in many types of CVD [29]. In addition, obesity (BMI Score) and physical activity have been shown to play an essential role in periodontitis and cardiovascular health [30]. The above evidence suggests that the CVH score, as measured by LE8, has the potential value to become a monitor of cardiovascular fitness level and prevalence and severity of periodontitis, and our results demonstrate the negative association of CVH with periodontitis from these perspectives.

Subgroup analyses of this study also elaborated on an important result: socioeconomic status modified the association between CVH and periodontitis, with participants with lower education and lower levels of household income elevating their CVH levels, resulting in a higher reduction in the odds of periodontitis than the rest of the group, so that participants with lower socioeconomic status should place more emphasis on improving or maintaining the different perspectives as measured by LE8 Cardiovascular health. There are several lines of evidence that low socioeconomic status increases the risk of cardiovascular and metabolic diseases [31–33]. Furthermore, the odds of periodontitis and disease severity have been reported to be negatively associated with socioeconomic status.

The mechanisms behind the negative correlation between cardiovascular health and the odds of periodontitis are extensive and complex. There are currently three main narratives explaining these mechanisms: (1) direct attachment of periodontal flora to endothelial cells through direct mechanisms and subsequent colonization of atherosclerotic plaques leading to plaque destabilization and atherosclerotic thrombotic events [5, 34]; (2) overlap of inflammatory pathways with higher systemic levels of inflammatory mediators [32, 35]; (3) shared clinical, environmental, and genetic risk factors [36–38].

Our study possesses several notable strengths. Foremost among them is the use of nationally representative data, which facilitates a comprehensive exploration of the association between cardiovascular health and the odds of periodontitis in the adult population of the United States [39]. This research is novel in examining the relationship between cardiovascular health scores, LE8, and periodontitis, offering new insights into the utility of CVH scores in the prevention and diagnosis of periodontitis. Nevertheless, certain limitations warrant acknowledgment. Firstly, the cross-sectional nature of the study design precludes the definitive establishment of causality [40, 41]. The reliance on self-reported data for certain indicators could potentially introduce bias. Furthermore, although we accounted for a broad spectrum of prevalent covariates to enhance the robustness of our findings, the constraints of the database meant that we could not completely eliminate the influence of all confounding factors on the results. An additional limitation of our study is the potential for selection bias. Individuals who refused participation in NHANES might differ fundamentally from those who participated, possibly influencing the generalizability of our findings. Moreover, the exclusion of older adults due to death before participation could bias our results, particularly in understanding the association of CVH and periodontitis in older populations. These factors underscore the need for cautious interpretation of our findings and suggest avenues for future research.

## Conclusion

In conclusion, our study reveals a negative association between CVH, as measured by LE8, and the odds of periodontitis. In this association, individuals of lower socioeconomic status would benefit more from maintaining higher levels of CVH to reduce the probability of periodontitis.

### Electronic supplementary material

Below is the link to the electronic supplementary material.

Supplementary Material 1

## Data Availability

The survey data are publicly available on the internet for data users and researchers throughout the world (www.cdc.gov/nchs/nhanes/).

## Abbreviations

CVD: Cardiovascular disease
CAL: Clinical Attachment Loss
PD: Probing Depth
AAP: American Academy of Periodontology
LE8: Life’s Essential 8
CVH: Cardiovascular health
AHA: American Heart Association
LS7: Life’s Simple 7
NHANES: National Health and Nutrition Examination Survey
NCHS: National Center for Health Statistics
BMI: Body mass index
DASH: Dietary Approaches to Stop Hypertension
SD: Standard deviation
RCS: Restricted cubic spline
